# Trimester-specific gestational weight gain and adverse outcomes in GDM women: a retrospective cohort study

**DOI:** 10.3389/fendo.2026.1861824

**Published:** 2026-06-30

**Authors:** Pei Yuan, Jing Huang, Jiangfan Wan, Lili Yu, Jialin Li, Bin Huang, Na Li, Hongwei Wei, Lin Kong, Jie Qin

**Affiliations:** 1 Network and Information Center, Maternal and Child Health Hospital of Guangxi Zhuang Autonomous Region, Nanning, China; 2 Department of Obstetrics, Maternal and Child Health Hospital of Guangxi Zhuang Autonomous Region, Nanning, China; 3 Department of Reproductive Medicine, The Reproductive Hospital of Guangxi Zhuang Autonomous Region, Nanning, China; 4 Birth Defects Prevention and Control Institute, Maternal and Child Health Hospital of Guangxi Zhuang Autonomous Region, Nanning, China; 5 Guangxi Key Laboratory of Birth Defects and Stem Cell Biobank, Nanning, China

**Keywords:** adverse pregnancy outcomes, generalized additive model (GAM), gestational diabetes mellitus, gestational weight gain, pre-pregnancy body mass index

## Abstract

**Aims:**

Gestational diabetes mellitus (GDM) is one of the most common metabolic disorders during pregnancy and is associated with an increased risk of multiple adverse pregnancy outcomes (APOs). Gestational weight gain (GWG) is an important indicator for disease monitoring and intervention; however, evidence regarding the associations between trimester-specific GWG patterns stratified by pre-pregnancy body mass index (BMI) and APOs among Chinese women with GDM remains limited.

**Materials and methods:**

Retrospective cohort study of 8, 562 singleton GDM pregnancies delivered at Maternal and Child Health Hospital of Guangxi Zhuang Autonomous Region (Feb 2021–Sep 2025). Women were classified into underweight, normal-weight, overweight, and obesity groups by pre-pregnancy BMI. GWG in early pregnancy, before Oral Glucose Tolerance Test (OGTT), and after OGTT was categorized as inadequate, adequate, or excessive per National Health Commission guidelines for GDM. Multivariable logistic regression with Benjamini–Hochberg false discovery rate (FDR) correction and generalized additive models (GAM) were used to assess associations and nonlinear relationships with APOs.

**Results:**

The associations between GWG and APOs differed significantly across pre-pregnancy BMI categories. Specifically, excessive GWG before OGTT diagnosis was associated with an increased risk of LGA among normal-weight and overweight women (aOR 1.71, 95% CI 1.38–2.13; and aOR 1.72, 95% CI 1.26–2.37, respectively). Excessive GWG after OGTT diagnosis was associated with an increased risk of preeclampsia (aOR 4.06, 95% CI 2.69–6.12; and aOR 2.49, 95% CI 1.46–4.24, respectively). In addition, excessive GWG before OGTT diagnosis was associated with a lower risk of SGA (aOR 0.39, 95% CI 0.25–0.62) in women with underweight. In women with obesity, no significant associations were observed between GWG and APOs, although the directional trends were generally consistent with those in the overweight group. GAM analyses further supported nonlinear associations between GWG and several APOs.

**Conclusion:**

Stage-specific GWG patterns in GDM pregnancies showed heterogeneous associations with APOs across pre-pregnancy BMI categories. These findings suggest that weight management strategies tailored to both pre-pregnancy BMI and gestational stage may help optimize pregnancy outcomes in women with GDM.

## 1 Introduction

Gestational diabetes mellitus (GDM) is one of the most prevalent metabolic disorders during pregnancy, and its global incidence has continued to rise over the past decade (1, 2). GDM imposes substantial short- and long-term burdens on maternal and offspring health, including increased risks of macrosomia, preterm birth, and preeclampsia (3, 4), as well as elevated long-term risks of type 2 diabetes in mothers and metabolic disorders in offspring (5, 6).

Following the diagnosis of GDM, clinical management primarily relies on dietary modification, physical activity interventions, and pharmacological treatment when necessary to achieve glycemic control targets (7–9). As a noninvasive and self-monitorable indicator, gestational weight gain (GWG) is closely associated with glycemic control and hyperglycemia-related adverse pregnancy outcomes (APOs) (10–12). In recent years, several studies have proposed recommended ranges of GWG specifically for women with GDM (13–15), and others have reported that both excessive and inadequate GWG increase the risk of APOs in this population (16, 17). However, GWG is influenced by multiple factors, including ethnicity, regional characteristics, and pre-pregnancy body mass index (BMI), and substantial interpopulation differences may exist. Therefore, evaluating the applicability of guideline-recommended GWG ranges in local GDM populations is crucial for improving clinical care quality and identifying opportunities to reduce GDM-related APOs.

Moreover, providing stage-specific GWG guidance across pregnancy aligns with the evolving paradigm of comprehensive GDM management. Although GDM is typically diagnosed in mid-pregnancy using the oral glucose tolerance test (OGTT), accumulating evidence suggests that metabolic abnormalities are already present in early pregnancy (9, 18, 19). Several clinical trials have demonstrated that interventions initiated only at 24–28 weeks of gestation do not effectively reduce adverse long-term health outcomes in offspring (20–22). One possible explanation is that critical multisystem fetal development begins early in pregnancy (23). Most existing studies have focused on total GWG and have not distinguished the heterogeneous effects of weight changes during early pregnancy, mid-pregnancy (before OGTT), and late pregnancy (after OGTT) on pregnancy outcomes (13, 17, 24). Stage-specific assessment of GWG may better capture the true risk patterns associated with weight gain. In addition, pre-pregnancy BMI is considered an important modifier of the relationship between GWG and pregnancy outcomes. Previous studies have shown that women who are underweight, normal weight, overweight, or obese differ in their sensitivity to weight gain, and the same amount of weight gain may be associated with different pregnancy risks (25, 26). Nevertheless, evidence regarding the associations between GWG at different gestational stages and APOs remains limited.

In December 2023, the National Health Commission (NHC) of the People’s Republic of China released the Standard of recommended gestational weight gain for women with gestational diabetes mellitus (27). This guideline, for the first time, proposed trimester-specific GWG reference ranges stratified by pre-pregnancy BMI, providing more actionable guidance for weight management among Chinese women with GDM. However, the applicability of these recommendations across different regions and specific populations has yet to be validated. Guangxi Zhuang Autonomous Region is located in the southern part of China, and is the settlement area of multiple minorities, particularly the Zhuang ethnic group. Based on the NHC recommendations, this study categorized stage-specific GWG as inadequate, adequate, or excessive and used these categories as the primary exposure variables to systematically evaluate their associations with APOs. Additionally, generalized additive models (GAM) were employed to construct smooth curves and explore potential nonlinear dose–response relationships. This study aimed to provide scientific evidence to support the development of more precise and individualized weight management strategies for women with GDM.

## 2 Materials and methods

### 2.1 Study population

**Figure 1:**
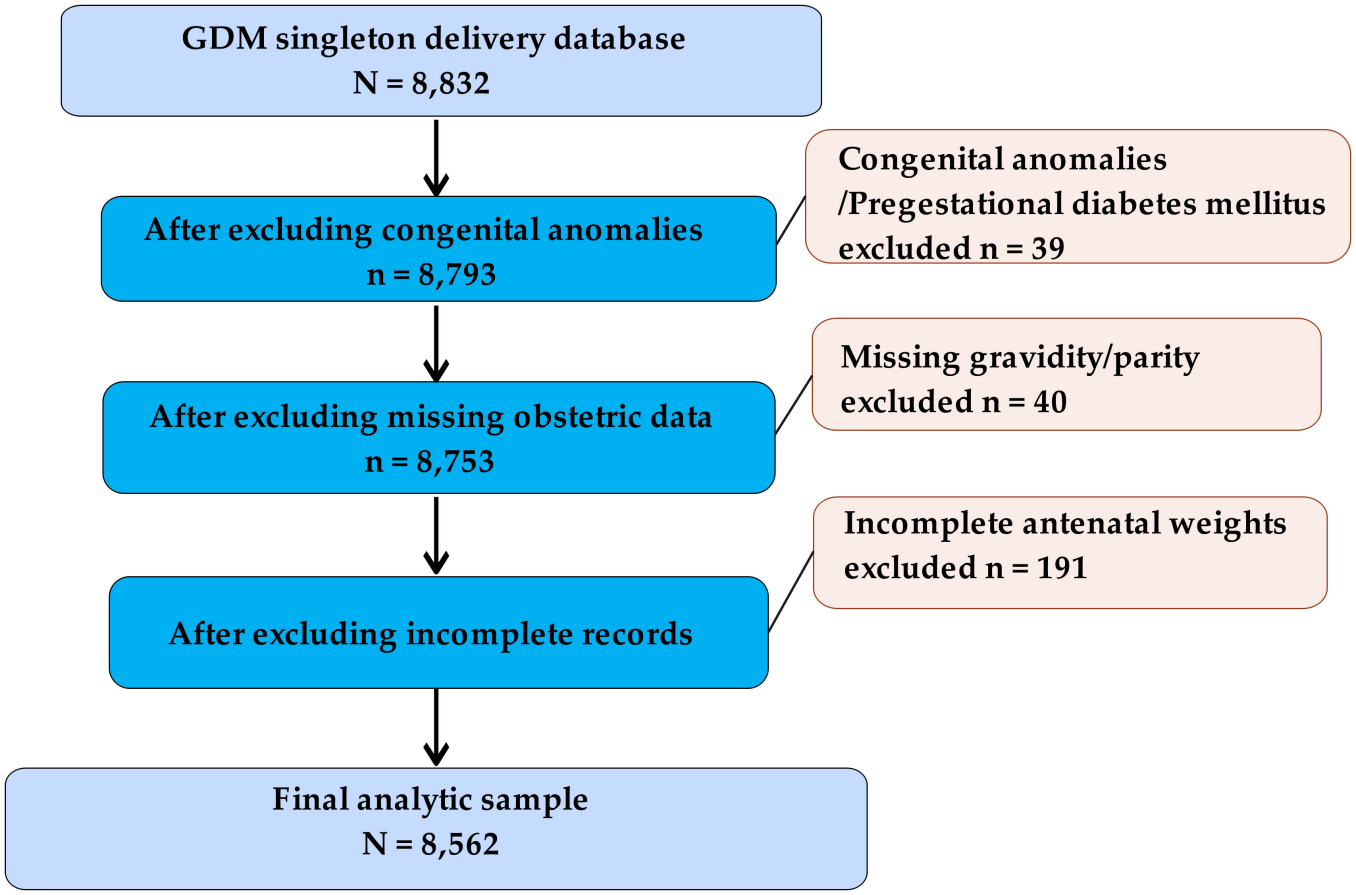
Study flow chart.

This retrospective cohort study included pregnant women who established prenatal care records and delivered at the two campuses of Maternal and Child Health Hospital of Guangxi Zhuang Autonomous Region between February 2021 and September 2025. The inclusion criteria were as follows: (1) singleton live birth; (2) diagnosis of GDM. The exclusion criteria were: (1) presence of congenital anomalies in the newborn; (2) pregestational diabetes mellitus; (3) incomplete clinical data. A total of 8, 562 pregnant women were ultimately included in the analysis (**Figure 1**). The study protocol complied with the Declaration of Helsinki and was approved by the Ethics Committee of the Maternal and Child Health Hospital of Guangxi Zhuang Autonomous Region [Approval No.: (2017) lun han shen di (4-2) hao)]. According to both of the national and institutional regulations, written informed consent for participation was waived for this retrospective study.

### 2.2 Data collection and definitions

Data were extracted from the electronic medical record system, including maternal age, gravidity, ethnicity, self-reported pre-pregnancy weight, height, and obstetric characteristics. Ethnicity was recorded according to the national identification system and categorized as Han, Zhuang, or other ethnic minority groups. Height and weight measurements were performed with participants barefoot and wearing light clothing, using regularly calibrated JUMPER medical digital instruments (#JPD-BS200, Shenzhen Jumper Medical Equipment Co., Ltd., China). Body weight was measured to the nearest 0.01 kg, and height was measured to the nearest 0.1 cm. Gestational age was initially calculated based on the first day of the last menstrual period and verified by early-pregnancy ultrasonography.

According to the Guidelines for the Prevention and Control of Overweight and Obesity in Chinese Adults (28), pre-pregnancy BMI (kg/m²) was categorized as underweight (BMI < 18.5), normal weight (18.5 ≤ BMI < 24.0), overweight (24.0 ≤ BMI < 28.0), and obese (BMI ≥ 28.0). The diagnosis of GDM followed the criteria recommended by the International Association of Diabetes and Pregnancy Study Groups (IADPSG) in 2010 (29) and was based on a 75-g oral glucose tolerance test (OGTT) performed at 24–28 weeks of gestation. GDM was diagnosed when any one of the following thresholds was met: fasting plasma glucose (FPG) ≥ 5.1 mmol/L; 1-hour postload glucose ≥ 10.0 mmol/L; or 2-hour postload glucose ≥ 8.5 mmol/L. Newborns with a birth weight < 2, 500 g within 1 hour after delivery were classified as having low birth weight (LBW), whereas those with a birth weight ≥ 4, 000 g were classified as macrosomia (30). The diagnostic criteria for preeclampsia were based on the guidelines issued by the Chinese Medical Association (31). In addition, according to local reference standards for Guangxi, newborns with birth weights above the 90th percentile for sex- and gestational age–specific distributions were defined as large for gestational age (LGA), and those below the 10th percentile were defined as small for gestational age (SGA) (32). Preterm birth was defined as delivery before 37 completed weeks of gestation (25).

Since clinical visit dates do not always coincide with specific gestational milestones, and guidelines require body weight values at particular dates for evaluation (27), cubic spline interpolation (using the na.spline function in the R ‘zoo’ package) (33) was applied to estimate weight at these required time points. Specifically, for participants without weight records on the exact required dates (13^+6^ weeks and the day of OGTT), available measurements within a ±2-week window were used to estimate these values.

### 2.3 Statistical analysis

GWG during early pregnancy, before OGTT diagnosis, and after OGTT diagnosis were calculated based on maternal weight measurements at the corresponding gestational weeks and were standardized using Z scores. Recommended GWG ranges are shown in **Supplementary Table 1**. Categorical variables were described as frequencies and percentages, whereas continuous variables were presented as means ± standard deviations or medians with interquartile ranges (IQR), depending on data distribution. Group comparisons were performed using one-way analysis of variance (ANOVA), Kruskal–Wallis test, or χ² test, as appropriate. Primary analyses were conducted separately within each pre-pregnancy BMI stratum. First, the distribution of different GWG patterns across adverse pregnancy outcomes was compared. To further explore nonlinear dose–response relationships, GAM were applied, with Z-score–standardized GWG rates entered as continuous independent variables to model smooth associations with multiple adverse pregnancy outcomes. Multivariable logistic regression models were used to evaluate the independent associations between GWG at different gestational stages and APOs. Benjamini-Hochberg false discovery rate (FDR) correction (34–36) was subsequently applied to account for multiple comparisons. A two-sided p value < 0.05 was considered nominally significant, whereas an FDR-adjusted q value < 0.05 was considered statistically significant after multiple-comparison correction. All statistical analyses were performed using R software (version 4.3.2).

## 3 Results

### 3.1 Characteristics of the study population

A total of 8, 562 pregnant women with GDM were included in this study. According to pre-pregnancy BMI, participants were categorized into groups of underweight (n = 599), normal weight (n = 5, 341), overweight (n = 2, 049), and obese (n = 573). Baseline demographic and clinical characteristics of the study population were summarized in **Table 1**. Maternal age differed significantly across pre-pregnancy BMI categories (*p* < 0.001). The proportion of advanced maternal age (≥35 years) was substantially higher in the overweight and obesity groups (46.8% and 46.2%, respectively), whereas the underweight group had the highest proportion of younger women (<25 years, 5.5%). Significant differences were also observed in ethnicity and place of residence among pre-pregnancy BMI groups (both *p* < 0.050). With respect to reproductive history, both gravidity and parity differed significantly across groups (both *p* < 0.001). The proportion of nulliparous women was highest in the underweight group (63.1%), whereas multiparity was more common in the overweight and obesity groups (approximately 56.9%–58.9%). Gestational weight gain during mid-pregnancy and late pregnancy (before OGTT, after OGTT), as well as total GWG, showed a significant decreasing trend with increasing pre-pregnancy BMI (*P* for trend < 0.001). Newborn birth weight increased significantly with increasing pre-pregnancy BMI (*p* < 0.001).

**Table 1:** Demographic and clinical characteristics of the study population .

Characteristic	Overall, N = 8, 562	Underweight, N = 599	Normal weight, N = 5, 341	Overweight, N = 2, 049	Obese, N = 573	p-value	P for trend
Age, n (%)						<0.001	<0.001
<25	145 (1.7)	33 (5.5)	77 (1.4)	27 (1.3)	8 (1.4)		
25-30	1, 515 (17.7)	174 (29.0)	958 (17.9)	290 (14.2)	93 (16.2)		
30-35	3, 538 (41.3)	255 (42.6)	2, 303 (43.1)	773 (37.7)	207 (36.1)		
≥35	3, 364 (39.3)	137 (22.9)	2, 003 (37.5)	959 (46.8)	265 (46.2)		
Ethnicity, n (%)						0.003	NA
Han	4, 641 (54.2)	354 (59.1)	2, 905 (54.4)	1, 106 (54.0)	276 (48.2)		
Zhuang	3, 549 (41.5)	231 (38.6)	2, 190 (41.0)	862 (42.1)	266 (46.4)		
Other ethnic minorities	372 (4.3)	14 (2.3)	246 (4.6)	81 (4.0)	31 (5.4)		
Residence, n (%)						0.016	NA
Rural	5, 796 (67.7)	424 (70.8)	3, 560 (66.7)	1, 432 (69.9)	380 (66.3)		
City	2, 766 (32.3)	175 (29.2)	1, 781 (33.3)	617 (30.1)	193 (33.7)		
Gravidity, n (%)						<0.001	<0.001
1	2, 355 (27.5)	238 (39.7)	1, 541 (28.9)	448 (21.9)	128 (22.3)		
≥2	6, 207 (72.5)	361 (60.3)	3, 800 (71.1)	1, 601 (78.1)	445 (77.7)		
Parity, n (%)						<0.001	<0.001
Primiparous	4, 190 (48.9)	378 (63.1)	2, 722 (51.0)	843 (41.1)	247 (43.1)		
Multiparous	4, 372 (51.1)	221 (36.9)	2, 619 (49.0)	1, 206 (58.9)	326 (56.9)		
Early-pregnancy GWG (kg)						0.017	0.514
Mean ± SD	1.61 ± 2.19	1.59 ± 1.78	1.66 ± 2.15	1.56 ± 2.31	1.35 ± 2.51		
GWG rate before OGTT (kg/week)						<0.001	<0.001
Mean ± SD	0.47 ± 0.17	0.51 ± 0.14	0.49 ± 0.16	0.43 ± 0.18	0.36 ± 0.19		
GWG rate after OGTT (kg/week)						<0.001	<0.001
Mean ± SD	0.33 ± 0.19	0.39 ± 0.17	0.33 ± 0.18	0.30 ± 0.19	0.30 ± 0.22		
GWG (kg)						<0.001	<0.001
Mean ± SD	11.35 ± 3.75	12.74 ± 3.10	11.71 ± 3.50	10.53 ± 4.06	9.44 ± 4.20		
Gestation age (weeks)						0.01	0.955
Mean ± SD	38.75 ± 1.22	38.77 ± 1.22	38.77 ± 1.20	38.75 ± 1.20	38.53 ± 1.46		
Infant gender, n (%)						0.42	NA
Girl	4, 005 (46.8)	263 (43.9)	2, 526 (47.3)	947 (46.2)	269 (46.9)		
Boy	4, 557 (53.2)	336 (56.1)	2, 815 (52.7)	1, 102 (53.8)	304 (53.1)		
Birth weight (g)						<0.001	<0.001
Mean ± SD	3, 159.91 ± 751.73	2, 990.46 ± 358.35	3, 129.17 ± 609.53	3, 265.82 ± 1, 121.41	3, 244.80 ± 515.78		
Preeclampsia, n (%)						<0.001	0.189
NO	8, 255 (96.4)	591 (98.7)	5, 214 (97.6)	1, 946 (95.0)	504 (88.0)		
YES	307 (3.6)	8 (1.3)	127 (2.4)	103 (5.0)	69 (12.0)		
Preterm birth, n (%)						0.004	0.556
NO	8, 028 (93.8)	560 (93.5)	5, 026 (94.1)	1, 925 (93.9)	517 (90.2)		
YES	534 (6.2)	39 (6.5)	315 (5.9)	124 (6.1)	56 (9.8)		
LBW, n (%)						0.033	0.113
NO	8, 076 (94.3)	555 (92.7)	5, 033 (94.2)	1, 954 (95.4)	534 (93.2)		
YES	486 (5.7)	44 (7.3)	308 (5.8)	95 (4.6)	39 (6.8)		
Macrosomia, n (%)						<0.001	0.076
NO	8, 335 (97.3)	596 (99.5)	5, 249 (98.3)	1, 957 (95.5)	533 (93.0)		
YES	227 (2.7)	3 (0.5)	92 (1.7)	92 (4.5)	40 (7.0)		
SGA, n (%)						<0.001	<0.001
NO	7, 396 (86.4)	460 (76.8)	4, 553 (85.2)	1, 861 (90.8)	522 (91.1)		
YES	1, 166 (13.6)	139 (23.2)	788 (14.8)	188 (9.2)	51 (8.9)		
LGA, n (%)						<0.001	<0.001
NO	7, 737 (90.4)	585 (97.7)	4, 924 (92.2)	1, 757 (85.7)	471 (82.2)		
YES	825 (9.6)	14 (2.3)	417 (7.8)	292 (14.3)	102 (17.8)		
Placental abruption, n (%)						0.055	0.734
NO	8, 309 (97.0)	578 (96.5)	5, 167 (96.7)	2, 002 (97.7)	562 (98.1)		
YES	253 (3.0)	21 (3.5)	174 (3.3)	47 (2.3)	11 (1.9)		
Postpartum hemorrhage, n (%)						0.928	0.523
NO	8, 345 (97.5)	586 (97.8)	5, 202 (97.4)	1, 998 (97.5)	559 (97.6)		
YES	217 (2.5)	13 (2.2)	139 (2.6)	51 (2.5)	14 (2.4)		

^1^ NA (not applicable).

Several adverse pregnancy outcomes differed markedly across BMI categories. The risks of preeclampsia, macrosomia, and LGA increased progressively with higher pre-pregnancy BMI (all *p* < 0.010). Notably, the incidence of preeclampsia reached 12.0% in the obesity group, compared with only 1.3% in the underweight group. In contrast, the incidence of SGA was highest in the underweight group (23.2%, *p* < 0.001). These findings suggest that pre-pregnancy BMI may play an important role in determining pregnancy outcomes.

### 3.2 Associations between stage-specific gestational weight gain patterns and adverse pregnancy outcomes by pre-pregnancy BMI category

As shown in **Table 2**, the distribution of the three GWG patterns (inadequate, adequate, and excessive) differed substantially across pre-pregnancy BMI categories at each gestational stage. Excessive GWG was more common before OGTT among women with overweight or obesity, whereas inadequate GWG became more frequent after OGTT, particularly among underweight women.

**Table 2:** Distribution of adverse pregnancy outcomes according to trimester-specific GWG categories stratified by pre-pregnancy BMI categories.

	Early Pregnancy				Before OGTT				After OGTT			
Underweight (n= 599)												
	Inadequate, N = 96 (16.0)	Adequate, N = 269 (44.9)	Excessive, N = 234 (39.1)	p-value	Inadequate, N = 95 (15.9)	Adequate, N = 263 (43.9)	Excessive, N = 241 (40.2)	p-value	Inadequate, N = 281 (46.9)	Adequate, N = 220 (36.7)	Excessive, N = 98 (16.4)	p-value
Preeclampsia	0 (0.0)	3 (1.1)	5 (2.1)	0.345	0 (0.0)	4 (1.5)	4 (1.7)	0.642	1 (0.4)	5 (2.3)	2 (2.0)	0.092
Preterm birth	6 (6.3)	18 (6.7)	15 (6.4)	0.986	13 (13.7)	15 (5.7)	11 (4.6)	0.007	20 (7.1)	14 (6.4)	5 (5.1)	0.780
LBW	7 (7.3)	20 (7.4)	17 (7.3)	0.997	11 (11.6)	22 (8.4)	11 (4.6)	0.060	23 (8.2)	13 (5.9)	8 (8.2)	0.590
Macrosomia	0 (0.0)	1 (0.4)	2 (0.9)	0.763	0 (0.0)	2 (0.8)	1 (0.4)	>0.999	1 (0.4)	2 (0.9)	0 (0.0)	0.757
SGA	27 (28.1)	61 (22.7)	51 (21.8)	0.448	29 (30.5)	76 (28.9)	34 (14.1)	<0.001	70 (24.9)	49 (22.3)	20 (20.4)	0.608
LGA	1 (1.0)	7 (2.6)	6 (2.6)	0.819	1 (1.1)	8 (3.0)	5 (2.1)	0.624	5 (1.8)	5 (2.3)	4 (4.1)	0.382
Placental abruption	7 (7.3)	8 (3.0)	6 (2.6)	0.113	6 (6.3)	8 (3.0)	7 (2.9)	0.286	11 (3.9)	6 (2.7)	4 (4.1)	0.727
Postpartum hemorrhage	4 (4.2)	5 (1.9)	4 (1.7)	0.380	2 (2.1)	5 (1.9)	6 (2.5)	0.930	5 (1.8)	7 (3.2)	1 (1.0)	0.479
Normal weight (n= 5, 341)												
	Inadequate, N = 1, 101 (20.6)	Adequate, N = 1, 985 (37.2)	Excessive, N = 2, 255 (42.2)	p-value	Inadequate, N = 424 (7.94)	Adequate, N = 2, 185 (40.9)	Excessive, N = 2, 732 (51.2)	p-value	Inadequate, N = 1, 821 (34.1)	Adequate, N = 2, 434 (45.6)	Excessive, N = 1, 086 (20.3)	p-value
Preeclampsia	23 (2.1)	47 (2.4)	57 (2.5)	0.735	10 (2.4)	55 (2.5)	62 (2.3)	0.851	25 (1.4)	38 (1.6)	64 (5.9)	<0.001
Preterm birth	64 (5.8)	116 (5.8)	135 (6.0)	0.972	35 (8.3)	117 (5.4)	163 (6.0)	0.066	117 (6.4)	113 (4.6)	85 (7.8)	<0.001
LBW	79 (7.2)	116 (5.8)	113 (5.0)	0.041	43 (10.1)	138 (6.3)	127 (4.6)	<0.001	110 (6.0)	124 (5.1)	74 (6.8)	0.107
Macrosomia	14 (1.3)	28 (1.4)	50 (2.2)	0.057	2 (0.5)	26 (1.2)	64 (2.3)	0.001	19 (1.0)	50 (2.1)	23 (2.1)	0.023
SGA	201 (18.3)	314 (15.8)	273 (12.1)	<0.001	80 (18.9)	368 (16.8)	340 (12.4)	<0.001	271 (14.9)	348 (14.3)	169 (15.6)	0.609
LGA	68 (6.2)	143 (7.2)	206 (9.1)	0.005	18 (4.2)	135 (6.2)	264 (9.7)	<0.001	115 (6.3)	200 (8.2)	102 (9.4)	0.007
Placental abruption	36 (3.3)	77 (3.9)	61 (2.7)	0.099	15 (3.5)	73 (3.3)	86 (3.1)	0.879	70 (3.8)	71 (2.9)	33 (3.0)	0.218
Postpartum hemorrhage	25 (2.3)	51 (2.6)	63 (2.8)	0.666	6 (1.4)	56 (2.6)	77 (2.8)	0.238	54 (3.0)	58 (2.4)	27 (2.5)	0.480
Overweight (n= 2, 049)												
	Inadequate, N = 506 (24.7)	Adequate, N = 702 (34.3)	Excessive, N = 841 (41.0)	p-value	Inadequate, N = 256 (12.5)	Adequate, N = 515 (25.1)	Excessive, N = 1, 278 (62.4)	p-value	Inadequate, N = 583 (28.5)	Adequate, N = 557 (27.2)	Excessive, N = 909 (44.4)	p-value
Preeclampsia	20 (4.0)	27 (3.8)	56 (6.7)	0.019	10 (3.9)	33 (6.4)	60 (4.7)	0.220	16 (2.7)	18 (3.2)	69 (7.6)	<0.001
Preterm birth	33 (6.5)	36 (5.1)	55 (6.5)	0.449	12 (4.7)	28 (5.4)	84 (6.6)	0.408	35 (6.0)	30 (5.4)	59 (6.5)	0.689
LBW	28 (5.5)	35 (5.0)	32 (3.8)	0.297	12 (4.7)	28 (5.4)	55 (4.3)	0.586	22 (3.8)	17 (3.1)	56 (6.2)	0.012
Macrosomia	20 (4.0)	29 (4.1)	43 (5.1)	0.519	7 (2.7)	15 (2.9)	70 (5.5)	0.021	18 (3.1)	21 (3.8)	53 (5.8)	0.028
SGA	56 (11.1)	65 (9.3)	67 (8.0)	0.161	27 (10.5)	61 (11.8)	100 (7.8)	0.020	55 (9.4)	44 (7.9)	89 (9.8)	0.461
LGA	62 (12.3)	95 (13.5)	135 (16.1)	0.124	21 (8.2)	55 (10.7)	216 (16.9)	<0.001	57 (9.8)	72 (12.9)	163 (17.9)	<0.001
Placental abruption	11 (2.2)	21 (3.0)	15 (1.8)	0.282	11 (4.3)	21 (4.1)	15 (1.2)	<0.001	17 (2.9)	14 (2.5)	16 (1.8)	0.320
Postpartum hemorrhage	11 (2.2)	14 (2.0)	26 (3.1)	0.337	11 (4.3)	11 (2.1)	29 (2.3)	0.138	15 (2.6)	11 (2.0)	25 (2.8)	0.644
Obese (n= 573)												
	Inadequate, N = 152 (26.5)	Adequate, N = 210 (36.6)	Excessive, N = 211 (36.8)	p-value	Inadequate, N = 82 (14.3)	Adequate, N = 130 (22.7)	Excessive, N = 361 (63.0)	p-value	Inadequate, N = 108 (18.8)	Adequate, N = 106 (18.5)	Excessive, N = 359 (62.7)	p-value
Preeclampsia	23 (15.1)	23 (11.0)	23 (10.9)	0.394	9 (11.0)	20 (15.4)	40 (11.1)	0.412	5 (4.6)	13 (12.3)	51 (14.2)	0.027
Preterm birth	15 (9.9)	20 (9.5)	21 (10.0)	0.988	13 (15.9)	15 (11.5)	28 (7.8)	0.062	9 (8.3)	7 (6.6)	40 (11.1)	0.329
LBW	13 (8.6)	12 (5.7)	14 (6.6)	0.567	9 (11.0)	13 (10.0)	17 (4.7)	0.033	8 (7.4)	4 (3.8)	27 (7.5)	0.389
Macrosomia	9 (5.9)	15 (7.1)	16 (7.6)	0.823	4 (4.9)	5 (3.8)	31 (8.6)	0.138	7 (6.5)	9 (8.5)	24 (6.7)	0.794
SGA	15 (9.9)	19 (9.0)	17 (8.1)	0.833	12 (14.6)	18 (13.8)	21 (5.8)	0.003	10 (9.3)	7 (6.6)	34 (9.5)	0.654
LGA	22 (14.5)	33 (15.7)	47 (22.3)	0.097	10 (12.2)	15 (11.5)	77 (21.3)	0.016	20 (18.5)	19 (17.9)	63 (17.5)	0.973
Placental abruption	4 (2.6)	3 (1.4)	4 (1.9)	0.691	1 (1.2)	2 (1.5)	8 (2.2)	>0.999	2 (1.9)	2 (1.9)	7 (1.9)	>0.999
Postpartum hemorrhage	4 (2.6)	5 (2.4)	5 (2.4)	>0.999	2 (2.4)	1 (0.8)	11 (3.0)	0.410	3 (2.8)	1 (0.9)	10 (2.8)	0.684

Distinct associations between trimester-specific GWG patterns and APOs were observed across pre-pregnancy BMI strata. In underweight women, inadequate GWG before OGTT was mainly associated with increased risks of preterm birth and SGA. Among women with normal weight, inadequate GWG was generally associated with higher risks of LBW and SGA, whereas excessive GWG was more frequently associated with macrosomia and LGA, particularly before and after OGTT. In women with overweight or obesity, excessive GWG was more consistently associated with elevated risks of preeclampsia and fetal overgrowth outcomes. Overall, GWG patterns before and after OGTT diagnosis showed the strongest associations with adverse pregnancy outcomes, whereas the impact of GWG during early pregnancy was relatively modest. Moreover, distinct risk patterns were observed across pre-pregnancy BMI categories, underscoring the necessity of BMI-stratified and stage-specific gestational weight management strategies.

### 3.3 Smoothed associations between Z-score–standardized gestational weight gain and adverse pregnancy outcomes

**Figure 2:**
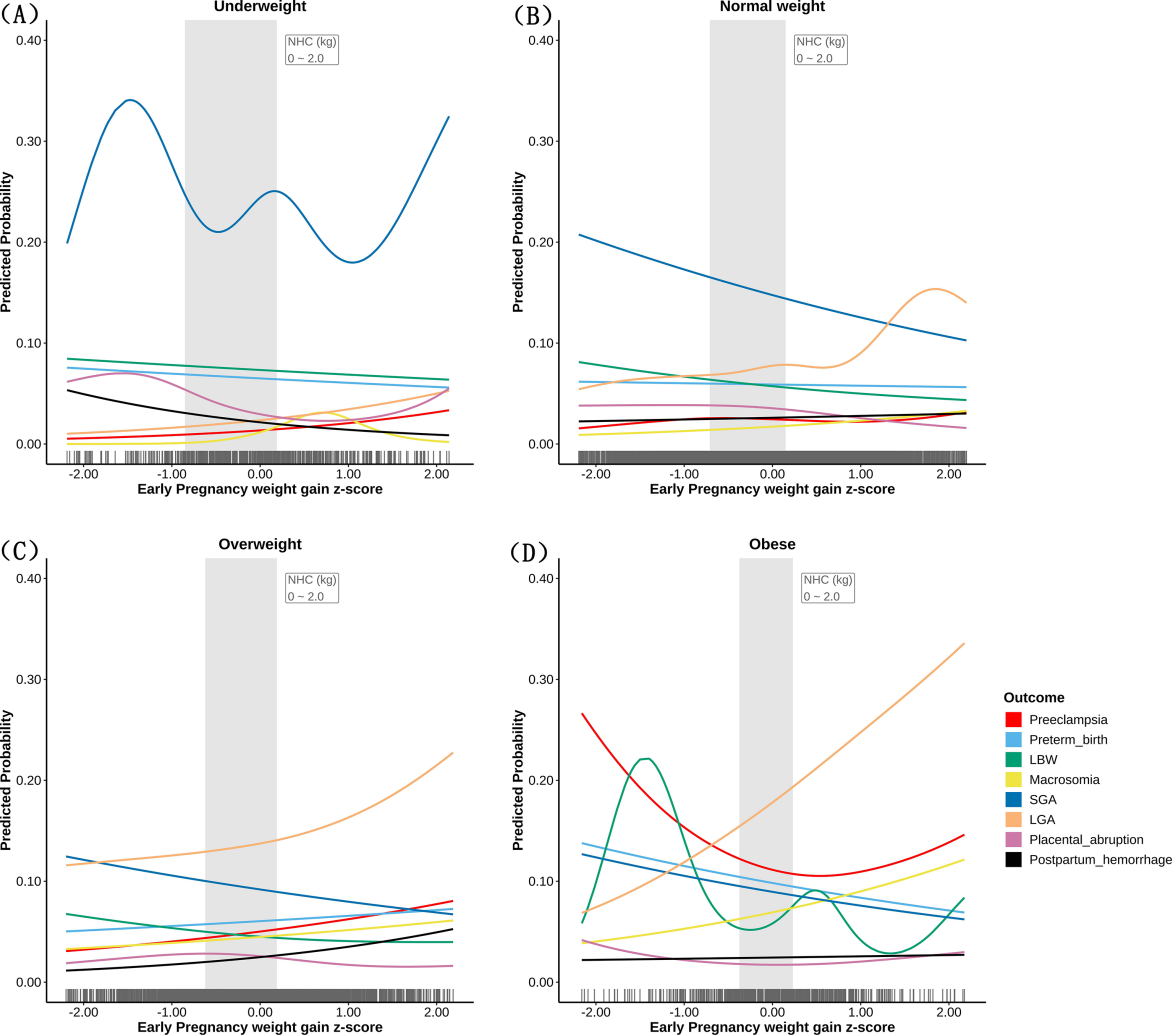
Smoothed associations between Z-score–standardized GWG in early pregnancy and APOs by pre-pregnancy BMI. **(A)** Underweight, **(B)** Normal weight, **(C)** Overweight, and **(D)** Obesity. Models were adjusted for age, ethnicity, residence, gravidity, and parity. Grey shaded areas indicate the recommended GWG range according to national guidelines. Vertical dashed lines represent the optimal GWG reference value.

**Figure 3:**
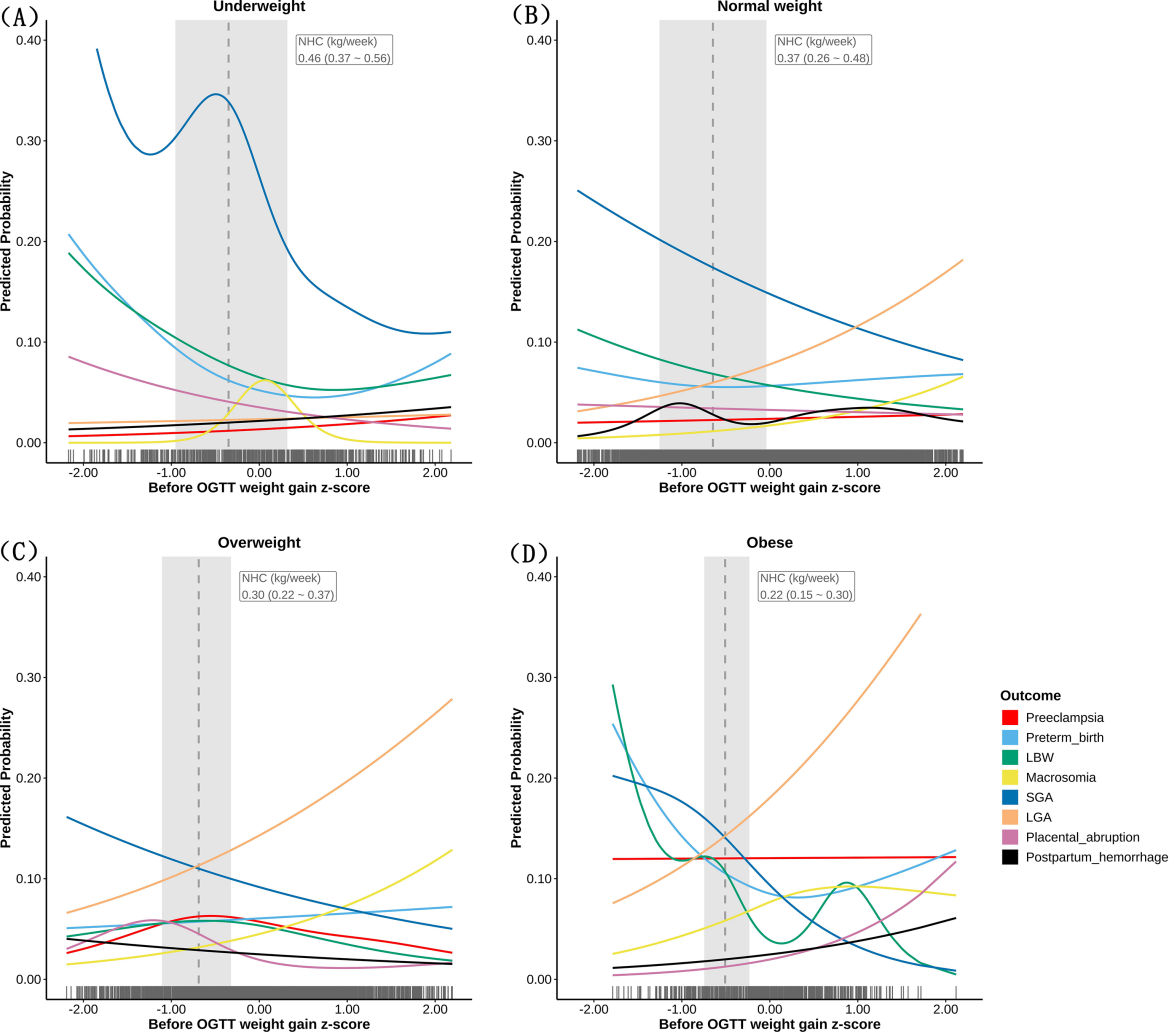
Smoothed associations between Z-score–standardized GWG before OGTT and APOs by pre-pregnancy BMI. All model adjustments, shaded areas, and reference lines are defined as in **Figure 2**.

**Figure 4:**
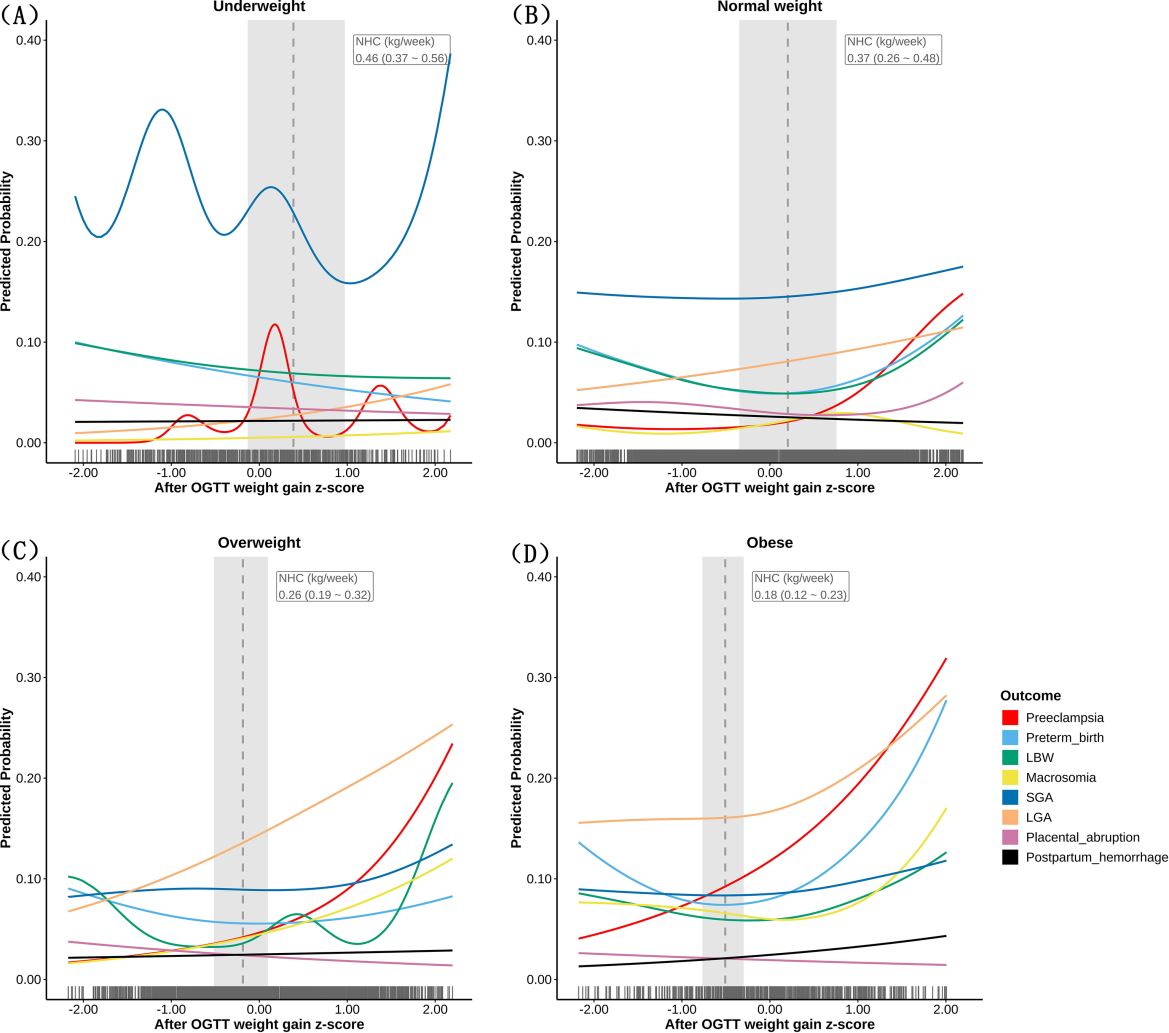
Smoothed associations between Z-score–standardized GWG after OGTT and APOs by pre-pregnancy BMI. All model adjustments, shaded areas, and reference lines are defined as in **Figure 2**.

**Figures 2**–**4** illustrates the smoothed associations between gestational weight gain during early pregnancy, before OGTT diagnosis, and after OGTT diagnosis, standardized using Z scores, and adverse pregnancy outcomes. For these models, the effective degrees of freedom (edf) and approximate p-values for each smooth term are reported in **Supplementary Table 3**, allowing for a detailed assessment of the statistical significance and nonlinearity of each smoothed association. Overall, consistent risk patterns were observed across pre-pregnancy BMI categories, whereby relatively lower weight gain rates were mainly associated with increased risks of outcomes related to insufficient fetal growth, whereas higher weight gain rates were primarily associated with increased risks of fetal overgrowth and preeclampsia; these associations were particularly pronounced in later pregnancy.

In the underweight group, as weight gain before OGTT diagnosis increased, the predicted probabilities of preterm birth, LBW, and SGA showed a decreasing trend. In women with normal weight and overweight before pregnancy, higher Z scores of weight gain before OGTT diagnosis were associated with lower predicted probabilities of SGA, while simultaneously showing a marked increase in the predicted probabilities of LGA and macrosomia. In the normal-weight group, the curves after OGTT diagnosis were the steepest, with each 1-SD increase in the Z score corresponding to a rapid increase in the predicted risks of preeclampsia and LGA, whereas preterm birth and LBW exhibited U-shaped relationships, with the lowest risks occurring within the NHC-recommended range of weight gain. In women with obesity, higher weight gain before OGTT diagnosis was associated with decreasing predicted probabilities of SGA and LBW, while the predicted probability of LGA increased. After OGTT diagnosis, increasing Z scores of weight gain were associated with significant upward trends in nearly all outcomes related to fetal overgrowth (LGA and macrosomia) as well as preeclampsia, suggesting that the primary risk in women with obesity arose from excessive weight gain. Weight gain within the NHC-recommended range, particularly after disease diagnosis, was consistently associated with lower incidences of adverse pregnancy outcomes.

The findings indicated that the direction of the associations between weight gain rates and pregnancy outcomes varied across different gestational stages, further emphasizing the importance of implementing individualized gestational weight management strategies under different pre-pregnancy BMI backgrounds.

### 3.4 Multivariable logistic regression analyses of gestational weight gain patterns and adverse pregnancy outcomes

To further evaluate the associations between stage-specific GWG and APOs, multivariable logistic regression analyses adjusted for age, ethnicity, residence, gravidity, and parity were performed (**Table 3**). Benjamini–Hochberg false discovery rate (FDR) correction was subsequently applied to account for multiple comparisons (**Supplementary Table 2**).

**Table 3:** Adjusted associations between trimester-specific GWG categories and adverse pregnancy outcomes stratified by pre-pregnancy BMI.

	Early Pregnancy		Before OGTT		After OGTT	
	Inadequate aOR (95% CI)	Excessive aOR (95% CI)	Inadequate aOR (95% CI)	Excessive aOR (95% CI)	Inadequate aOR (95% CI)	Excessive aOR (95% CI)
Underweight						
Preeclampsia	–	2.01 (0.46, 8.67)	–	0.95 (0.23, 3.99)	0.16 (0.02, 1.38)	1.07 (0.19, 5.97)
Preterm birth	0.96 (0.36, 2.51)	0.98 (0.48, 2.00)	2.67 (1.21, 5.91)^a^	0.73 (0.32, 1.63)	1.12 (0.55, 2.30)	0.72 (0.25, 2.07)
LBW	0.98 (0.40, 2.43)	0.96 (0.49, 1.89)	1.44 (0.66, 3.11)	0.54 (0.26, 1.15)	1.36 (0.67, 2.78)	1.39 (0.55, 3.50)
Macrosomia	–	2.33 (0.20, 26.81)	–	0.52 (0.04, 6.08)	0.35 (0.03, 4.01)	–
SGA	1.37 (0.80, 2.34)	0.98 (0.64, 1.51)	1.05 (0.62, 1.76)	0.39 (0.25, 0.62)^ce^	1.15 (0.75, 1.77)	0.88 (0.49, 1.59)
LGA	0.42 (0.05, 3.53)	0.92 (0.30, 2.83)	0.35 (0.04, 2.92)	0.73 (0.23, 2.32)	0.69 (0.19, 2.46)	2.12 (0.53, 8.37)
Placental abruption	2.98 (1.03, 8.66)^a^	0.87 (0.29, 2.56)	2.07 (0.69, 6.23)	1.01 (0.35, 2.87)	1.48 (0.53, 4.16)	1.67 (0.45, 6.16)
Postpartum hemorrhage	2.66 (0.68, 10.40)	0.96 (0.25, 3.68)	1.04 (0.20, 5.51)	1.48 (0.44, 5.03)	0.51 (0.16, 1.66)	0.33 (0.04, 2.75)
Normal weight						
Preeclampsia	0.88 (0.53, 1.45)	1.02 (0.69, 1.51)	0.91 (0.46, 1.80)	0.91 (0.63, 1.31)	0.83 (0.50, 1.39)	4.06 (2.69, 6.12)^ce^
Preterm birth	1.02 (0.74, 1.40)	1.00 (0.77, 1.29)	1.54 (1.04, 2.28)^a^	1.16 (0.91, 1.48)	1.37 (1.05, 1.79)^a^	1.79 (1.34, 2.40)ce
LBW	1.23 (0.91, 1.65)	0.84 (0.65, 1.10)	1.71 (1.19, 2.45)^bd^	0.72 (0.56, 0.92)^bd^	1.19 (0.91, 1.55)	1.35 (1.00, 1.82)^a^
Macrosomia	0.92 (0.48, 1.76)	1.55 (0.97, 2.48)	0.37 (0.09, 1.58)	2.06 (1.30, 3.27)^bd^	0.49 (0.29, 0.84)^bd^	1.05 (0.64, 1.74)
SGA	1.15 (0.94, 1.40)	0.73 (0.61, 0.87)^ce^	1.20 (0.91, 1.57)	0.67 (0.57, 0.79)^ce^	1.04 (0.88, 1.24)	1.09 (0.89, 1.33)
LGA	0.89 (0.66, 1.20)	1.27 (1.02, 1.59)^a^	0.63 (0.38, 1.05)	1.71 (1.38, 2.13)^ce^	0.73 (0.58, 0.93)^a^	1.20 (0.93, 1.55)
Placental abruption	0.85 (0.57, 1.27)	0.68 (0.48, 0.96)^a^	1.05 (0.60, 1.86)	0.97 (0.70, 1.33)	1.32 (0.94, 1.85)	1.07 (0.70, 1.63)
Postpartum hemorrhage	0.87 (0.54, 1.42)	1.08 (0.74, 1.57)	0.55 (0.24, 1.29)	1.10 (0.78, 1.57)	1.23 (0.84, 1.79)	1.04 (0.65, 1.66)
Overweight						
Preeclampsia	1.06 (0.58, 1.91)	1.75 (1.09, 2.81)^a^	0.65 (0.32, 1.36)	0.71 (0.46, 1.11)	0.89 (0.45, 1.77)	2.49 (1.46, 4.24)^cd^
Preterm birth	1.28 (0.79, 2.09)	1.25 (0.81, 1.92)	0.85 (0.42, 1.71)	1.23 (0.79, 1.92)	1.12 (0.68, 1.85)	1.24 (0.79, 1.96)
LBW	1.14 (0.68, 1.90)	0.72 (0.44, 1.17)	0.90 (0.45, 1.80)	0.77 (0.48, 1.23)	1.28 (0.67, 2.44)	2.14 (1.22, 3.72)^b^
Macrosomia	0.93 (0.52, 1.67)	1.21 (0.75, 1.97)	0.89 (0.36, 2.22)	1.96 (1.11, 3.46)^a^	0.79 (0.41, 1.49)	1.65 (0.98, 2.77)
SGA	1.26 (0.86, 1.84)	0.83 (0.58, 1.20)	0.95 (0.58, 1.54)	0.63 (0.45, 0.88)^b^	1.27 (0.84, 1.93)	1.26 (0.86, 1.84)
LGA	0.88 (0.62, 1.24)	1.21 (0.91, 1.61)	0.71 (0.42, 1.20)	1.72 (1.26, 2.37)^cd^	0.71 (0.49, 1.02)	1.53 (1.13, 2.06)b
Placental abruption	0.70 (0.33, 1.47)	0.57 (0.29, 1.11)	1.00 (0.47, 2.12)	0.28 (0.14, 0.55)^ce^	1.12 (0.54, 2.29)	0.71 (0.34, 1.47)
Postpartum hemorrhage	1.09 (0.49, 2.43)	1.55 (0.80, 2.99)	2.01 (0.86, 4.73)	1.07 (0.53, 2.16)	1.35 (0.61, 2.97)	1.50 (0.73, 3.08)
Obese						
Preeclampsia	1.52 (0.81, 2.85)	0.96 (0.51, 1.79)	0.67 (0.28, 1.57)	0.68 (0.38, 1.23)	0.33 (0.11, 0.98)^a^	1.23 (0.64, 2.39)
Preterm birth	1.11 (0.55, 2.28)	1.02 (0.53, 1.97)	1.49 (0.66, 3.38)	0.66 (0.34, 1.29)	1.31 (0.47, 3.69)	1.71 (0.74, 3.98)
LBW	1.73 (0.75, 4.00)	1.33 (0.59, 3.02)	1.21 (0.48, 3.10)	0.43 (0.20, 0.93)^a^	2.07 (0.59, 7.23)	1.77 (0.59, 5.26)
Macrosomia	0.84 (0.35, 2.00)	1.08 (0.51, 2.28)	1.25 (0.32, 4.83)	2.43 (0.92, 6.44)	0.72 (0.25, 2.02)	0.70 (0.31, 1.58)
SGA	1.21 (0.59, 2.50)	0.97 (0.48, 1.96)	1.24 (0.55, 2.81)	0.38 (0.19, 0.75)^b^	1.46 (0.52, 4.05)	1.37 (0.58, 3.23)
LGA	0.86 (0.48, 1.57)	1.56 (0.94, 2.59)	0.99 (0.42, 2.35)	2.04 (1.12, 3.72)^a^	1.01 (0.50, 2.04)	0.97 (0.55, 1.73)
Placental abruption	2.01 (0.44, 9.30)	1.45 (0.31, 6.72)	0.87 (0.08, 9.99)	1.52 (0.32, 7.31)	1.08 (0.15, 7.85)	1.05 (0.21, 5.23)
Postpartum hemorrhage	1.07 (0.27, 4.16)	1.15 (0.31, 4.31)	3.51 (0.30, 41.21)	4.07 (0.51, 32.27)	3.02 (0.30, 30.84)	3.23 (0.40, 26.14)

^1^ Models were adjusted for age, ethnicity, residence, gravidity, and parity.

^2^ a: *p* < 0.05, b: *p* < 0.01, c: *p* < 0.001.

^3^ d: *q* < 0.05; e: *q* < 0.01; f: *q* < 0.001 (BH-FDR corrected).

^4^ The ‘-’ indicates not estimable due to zero events; see **Table 2** for raw counts.

Consistent with the GAM analyses, associations related to fetal growth outcomes were primarily observed before OGTT diagnosis. In the normal-weight and overweight groups, excessive GWG before OGTT diagnosis was primarily associated with fetal growth–related outcomes, including increased risks of macrosomia and LGA in the normal-weight group (aOR 2.06, 95% CI 1.30–3.27, q = 0.039; aOR 1.71, 95% CI 1.38–2.13, q = 0.004, respectively) and increased LGA risk in the overweight group (aOR 1.72, 95% CI 1.26–2.37, q = 0.017). Excessive GWG before OGTT diagnosis was also associated with lower SGA risk in the normal-weight group (aOR 0.67, 95% CI 0.57–0.79, q = 0.004).

In contrast, excessive GWG after OGTT diagnosis was mainly associated with maternal and preterm outcomes. Excessive GWG after OGTT diagnosis was associated with increased risks of preeclampsia in both the normal-weight and overweight groups (aOR 4.06, 95% CI 2.69–6.12, q = 0.004; aOR 2.49, 95% CI 1.46–4.24, q = 0.017, respectively). In addition, excessive GWG after OGTT diagnosis was associated with an increased risk of preterm birth in the normal-weight group. In the underweight group, the only robust association was between excessive GWG before OGTT diagnosis and lower SGA risk (aOR 0.39, 95% CI 0.25–0.62, q = 0.004).

In the obesity group, none of the associations remained statistically significant after FDR correction. However, several nominal associations showed directional consistency with those observed in the overweight group. For example, inadequate GWG after OGTT diagnosis was associated with a lower risk of preeclampsia (aOR 0.33, 95% CI 0.11–0.98, p < 0.05, q = 0.290), whereas excessive GWG before OGTT diagnosis showed trends toward lower risks of SGA and LBW, together with a higher risk of LGA.

Taken together, significant associations between GWG and APOs manifest both before and after OGTT diagnosis. Excessive weight gain relative to the NHC-recommended range is robustly associated with LGA, preeclampsia, and preterm birth, with the specific impact varying by pre-pregnancy BMI.

## 4 Discussion

Based on a large cohort of women with GDM, we systematically evaluated the associations between stage-specific GWG and APOs according to the NHC-recommended GWG targets. After FDR correction for multiple comparisons, distinct GWG–APO patterns remained evident across pre-pregnancy BMI categories and gestational stages. Associations related to fetal growth outcomes were primarily observed before OGTT diagnosis. Excessive GWG before OGTT diagnosis was consistently associated with higher risks of LGA and lower risks of SGA among women with normal weight and those with overweight. In contrast, excessive GWG after OGTT diagnosis was more strongly associated with maternal outcomes, particularly preeclampsia and preterm birth in these two groups. Among women with underweight, excessive GWG before OGTT diagnosis was associated with a lower risk of SGA. These findings underscore the importance of individualized GWG management strategies for women with GDM according to both pre-pregnancy BMI and gestational stage.

Compared with previous studies that applied the Institute of Medicine (IOM) guidelines, the present study adopted the 2023 NHC guidelines for GWG in women with GDM (27), which are considered more appropriate for Asian body composition. Several studies have shown that the IOM guidelines may overestimate the upper limit of GWG in Chinese women with obesity, thereby increasing the risk of excessive weight gain (30).

Previous studies based on total GWG or weight gain during mid-to-late pregnancy have reported that excessive weight gain is associated with increased risks of LGA, macrosomia, and preeclampsia, whereas inadequate weight gain is associated with increased risks of SGA, LBW, and preterm birth (10–13). The NHC guidelines recommend categorizing GWG in women with GDM into early pregnancy, before GDM diagnosis, and after GDM diagnosis, reflecting the unique metabolic characteristics of GDM compared with normal pregnancy and aligning more closely with clinical practice. Our findings suggest that GWG before OGTT diagnosis was more strongly associated with outcomes related to fetal growth, whereas GWG after OGTT diagnosis was more closely associated with maternal outcomes, particularly preeclampsia and preterm birth. These stage-specific patterns may help identify potential intervention windows for GWG management in women with GDM.

Among women with normal weight, excessive GWG before OGTT diagnosis was associated with increased risks of macrosomia and LGA, alongside a reduced risk of SGA. These findings are consistent with reports by Huang et al. (13) and Zheng et al. (26), further supporting the association between weight gain prior to GDM diagnosis and fetal growth trajectories. Notably, excessive GWG after OGTT diagnosis remained consistently associated with an increased risk of preeclampsia among both women with normal weight and those with overweight, aligning with previous evidence (10, 37, 38). Mechanistically, excessive weight gain during late pregnancy may aggravate insulin resistance, endothelial dysfunction, and oxidative stress, thereby contributing to the development of preeclampsia (39–41). Our findings suggest that the period after OGTT diagnosis may represent an important intervention window for maternal complication management in these populations. Among women with overweight, beyond the association with preeclampsia, excessive GWG before OGTT diagnosis was also associated with a higher risk of LGA and a lower risk of placental abruption, further supporting previous evidence that excessive GWG among women with GDM and higher pre-pregnancy BMI may be associated with increased risks of adverse pregnancy outcomes (17, 25, 30).

Statistical precision was limited in some BMI categories because of smaller sample sizes. Among women with obesity (n = 573), no associations survived FDR correction. However, several nominal associations (p < 0.05) mirrored findings in the overweight group: excessive GWG before OGTT diagnosis was associated with higher LGA risk and lower SGA risk, whereas inadequate GWG after OGTT diagnosis was associated with lower preeclampsia risk. These patterns suggest potentially shared association patterns, although the available evidence remains insufficient to determine whether the current NHC recommended GWG targets are fully applicable to this subgroup. Conversely, among women with underweight, excessive GWG before OGTT diagnosis was the only association that remained significant after FDR correction and was associated with a lower risk of SGA. Given that this subgroup had the highest baseline incidence of SGA (23.2%), these findings highlight the potential importance of adequate GWG before OGTT diagnosis in reducing the risk of fetal growth restriction among underweight women with GDM (33).

The stage-specific and BMI-stratified differences observed in this study may reflect dynamic changes in maternal metabolic status, placental function, and fetal growth regulation across different stages of pregnancy. Early pregnancy and the period before OGTT diagnosis represent critical phases for placental development and the gradual establishment of insulin resistance. Previous studies have shown that rapid weight gain during early pregnancy is primarily attributable to adipose tissue accumulation, which can increase inflammatory cytokine levels and disrupt adipokine balance, thereby accelerating the development of insulin resistance (9, 18, 19). In women at high risk of GDM, such metabolic alterations may exert long-term effects on placental blood flow and fetal nutrient supply even before OGTT diagnosis, which may explain the observed associations between excessive weight gain before OGTT diagnosis and increased risks of macrosomia and LGA (4, 14). After OGTT diagnosis, excessive weight gain may further amplify placental hormone–induced physiological insulin resistance, exacerbate endothelial dysfunction and oxidative stress, and consequently increase the risk of preeclampsia (42, 43). This process may, in turn, restrict placental nutrient delivery and induce fetal growth restriction (LBW) and preterm birth (44–46). These physiological mechanisms may help explain our findings that excessive GWG after OGTT diagnosis was associated with increased risks of preeclampsia among women with normal weight and overweight. Collectively, these findings suggest that the clinical impact of GWG may vary according to pre-pregnancy metabolic status and the physiological characteristics of different gestational stages.

Nevertheless, several limitations of this study should be acknowledged. First, this was a single-center study based on data from a provincial maternal and child health hospital in Guangxi, which may limit the generalizability of the findings to other regions or populations in China. Second, pre-pregnancy weight was self-reported and therefore subject to potential recall bias. Third, due to data limitations, potential confounders such as HbA1c, post-diagnosis fasting glucose and GDM treatments were not included. Finally, given the multiple stratified comparisons performed in this study, some subgroup findings should be interpreted cautiously despite the application of FDR correction for multiple testing. Further validation in independent prospective cohorts is warranted to confirm the observed associations.

## 5 Conclusions

The present study demonstrated substantial heterogeneity in the associations between gestational weight gain and adverse pregnancy outcomes across different pre-pregnancy BMI strata and gestational stages among women with GDM. Excessive GWG before and after OGTT diagnosis was associated with increased risks of several adverse pregnancy outcomes among normal-weight and overweight women. These findings support the application of the NHC-recommended GWG targets in the regional GDM population and further highlight the importance of individualized GWG management strategies tailored to both pre-pregnancy BMI and gestational stage. In addition, the findings in underweight women suggest that the applicability of current GWG targets in this subgroup may warrant further investigation.

## Data Availability

The dataset in this study is available from the corresponding author upon reasonable request. Requests to access the dataset should be directed to Jie Qin, watermelonjie83@gmail.com.
