# Supplementary material for: Trimester-specific gestational weight gain and adverse outcomes in GDM women: a retrospective cohort study
Source: Front Endocrinol (Lausanne). 2026 Jun 30;17:1861824. doi: 10.3389/fendo.2026.1861824 (PMC13364531; doi:10.3389/fendo.2026.1861824)
Supplement: Supplementary file 1 [file Table1.docx]

#### Supplementary Material

##### Table S1. Standard of recommended gestational weight gain for women with gestational diabetes mellitus.

| **Pre-Pregnancy BMI** | **Early-pregnancy GWG (kg)** | **GWG rate before OGTT (kg/week)** | **GWG rate after OGTT (kg/week)** |
| --- | --- | --- | --- |
| Underweight (BMI＜18.5 kg/m^2^) | 0 – 2.0 | 0.46(0.37～0.56) | 0.46(0.37～0.56) |
| Normal weight (18.5≤BMI＜24.0 kg/m^2^) | 0 – 2.0 | 0.37(0.26～0.48) | 0.37(0.26～0.48) |
| Overweight (24.0≤BMI＜28.0 kg/m^2^) | 0 – 2.0 | 0.30(0.22～0.37) | 0.26(0.19～0.32) |
| Obese (BMI≥28.0 kg/m^2^) | 0 – 2.0 | 0.22(0.15～0.30) | 0.18(0.12～0.23) |

##### Table S2. Results of multiple testing adjustment using the Benjamini-Hochberg (FDR) procedure for the association between GWG and adverse outcomes

| **Pre-pregnancy BMI** | **Outcome** | **Gestational**  **stage** | **GWG category**  **(vs Adequate)** | **aOR**  **(95% CI)** | **Unadjusted p-value** | **BH-FDR adjusted q-value** | **Sig.**  **(unadjusted)** | **Sig.**  **(FDR)** |
| --- | --- | --- | --- | --- | --- | --- | --- | --- |
| Underweight | Preeclampsia | early pregnancy | Inadequate | - | — | — | NA | NA |
| Underweight | Preeclampsia | early pregnancy | Excessive | 2.01 (0.46, 8.67) | 0.352 | 0.742 |  |  |
| Underweight | Preterm birth | early pregnancy | Inadequate | 0.96 (0.36, 2.51) | 0.930 | 0.996 |  |  |
| Underweight | Preterm birth | early pregnancy | Excessive | 0.98 (0.48, 2.00) | 0.949 | 0.996 |  |  |
| Underweight | LBW | early pregnancy | Inadequate | 0.98 (0.40, 2.43) | 0.970 | 0.996 |  |  |
| Underweight | LBW | early pregnancy | Excessive | 0.96 (0.49, 1.89) | 0.900 | 0.996 |  |  |
| Underweight | Macrosomia | early pregnancy | Inadequate | - | — | — | NA | NA |
| Underweight | Macrosomia | early pregnancy | Excessive | 2.33 (0.20, 26.81) | 0.496 | 0.822 |  |  |
| Underweight | SGA | early pregnancy | Inadequate | 1.37 (0.80, 2.34) | 0.254 | 0.699 |  |  |
| Underweight | SGA | early pregnancy | Excessive | 0.98 (0.64, 1.51) | 0.932 | 0.996 |  |  |
| Underweight | LGA | early pregnancy | Inadequate | 0.42 (0.05, 3.53) | 0.425 | 0.809 |  |  |
| Underweight | LGA | early pregnancy | Excessive | 0.92 (0.30, 2.83) | 0.885 | 0.996 |  |  |
| Underweight | Placental abruption | early pregnancy | Inadequate | 2.98 (1.03, 8.66) | **0.044** | 0.290 | * |  |
| Underweight | Placental abruption | early pregnancy | Excessive | 0.87 (0.29, 2.56) | 0.795 | 0.996 |  |  |
| Underweight | Postpartum hemorrhage | early pregnancy | Inadequate | 2.66 (0.68, 10.40) | 0.160 | 0.641 |  |  |
| Underweight | Postpartum hemorrhage | early pregnancy | Excessive | 0.96 (0.25, 3.68) | 0.958 | 0.996 |  |  |
| Underweight | Preeclampsia | before OGTT | Inadequate | - | — | — | NA | NA |
| Underweight | Preeclampsia | before OGTT | Excessive | 0.95 (0.23, 3.99) | 0.949 | 0.996 |  |  |
| Underweight | Preterm birth | before OGTT | Inadequate | 2.67 (1.21, 5.91) | **0.015** | 0.152 | * |  |
| Underweight | Preterm birth | before OGTT | Excessive | 0.73 (0.32, 1.63) | 0.442 | 0.809 |  |  |
| Underweight | LBW | before OGTT | Inadequate | 1.44 (0.66, 3.11) | 0.360 | 0.742 |  |  |
| Underweight | LBW | before OGTT | Excessive | 0.54 (0.26, 1.15) | 0.111 | 0.508 |  |  |
| Underweight | Macrosomia | before OGTT | Inadequate | - | — | — | NA | NA |
| Underweight | Macrosomia | before OGTT | Excessive | 0.52 (0.04, 6.08) | 0.603 | 0.883 |  |  |
| Underweight | SGA | before OGTT | Inadequate | 1.05 (0.62, 1.76) | 0.859 | 0.996 |  |  |
| Underweight | SGA | before OGTT | Excessive | 0.39 (0.25, 0.62) | **<0.001** | **0.004** | *** | †† |
| Underweight | LGA | before OGTT | Inadequate | 0.35 (0.04, 2.92) | 0.335 | 0.742 |  |  |
| Underweight | LGA | before OGTT | Excessive | 0.73 (0.23, 2.32) | 0.593 | 0.883 |  |  |
| Underweight | Placental abruption | before OGTT | Inadequate | 2.07 (0.69, 6.23) | 0.194 | 0.641 |  |  |
| Underweight | Placental abruption | before OGTT | Excessive | 1.01 (0.35, 2.87) | 0.990 | 0.998 |  |  |
| Underweight | Postpartum hemorrhage | before OGTT | Inadequate | 1.04 (0.20, 5.51) | 0.966 | 0.996 |  |  |
| Underweight | Postpartum hemorrhage | before OGTT | Excessive | 1.48 (0.44, 5.03) | 0.527 | 0.850 |  |  |
| Underweight | Preeclampsia | after OGTT | Inadequate | 0.16 (0.02, 1.38) | 0.094 | 0.477 |  |  |
| Underweight | Preeclampsia | after OGTT | Excessive | 1.07 (0.19, 5.97) | 0.943 | 0.996 |  |  |
| Underweight | Preterm birth | after OGTT | Inadequate | 1.12 (0.55, 2.30) | 0.753 | 0.982 |  |  |
| Underweight | Preterm birth | after OGTT | Excessive | 0.72 (0.25, 2.07) | 0.541 | 0.857 |  |  |
| Underweight | LBW | after OGTT | Inadequate | 1.36 (0.67, 2.78) | 0.395 | 0.781 |  |  |
| Underweight | LBW | after OGTT | Excessive | 1.39 (0.55, 3.50) | 0.488 | 0.822 |  |  |
| Underweight | Macrosomia | after OGTT | Inadequate | 0.35 (0.03, 4.01) | 0.397 | 0.781 |  |  |
| Underweight | Macrosomia | after OGTT | Excessive | - | — | — | NA | NA |
| Underweight | SGA | after OGTT | Inadequate | 1.15 (0.75, 1.77) | 0.509 | 0.835 |  |  |
| Underweight | SGA | after OGTT | Excessive | 0.88 (0.49, 1.59) | 0.669 | 0.921 |  |  |
| Underweight | LGA | after OGTT | Inadequate | 0.69 (0.19, 2.46) | 0.568 | 0.883 |  |  |
| Underweight | LGA | after OGTT | Excessive | 2.12 (0.53, 8.37) | 0.286 | 0.712 |  |  |
| Underweight | Placental abruption | after OGTT | Inadequate | 1.48 (0.53, 4.16) | 0.453 | 0.809 |  |  |
| Underweight | Placental abruption | after OGTT | Excessive | 1.67 (0.45, 6.16) | 0.440 | 0.809 |  |  |
| Underweight | Postpartum hemorrhage | after OGTT | Inadequate | 0.51 (0.16, 1.66) | 0.265 | 0.706 |  |  |
| Underweight | Postpartum hemorrhage | after OGTT | Excessive | 0.33 (0.04, 2.75) | 0.304 | 0.742 |  |  |
| Normal weight | Preeclampsia | early pregnancy | Inadequate | 0.88 (0.53, 1.45) | 0.609 | 0.883 |  |  |
| Normal weight | Preeclampsia | early pregnancy | Excessive | 1.02 (0.69, 1.51) | 0.919 | 0.996 |  |  |
| Normal weight | Preterm birth | early pregnancy | Inadequate | 1.02 (0.74, 1.40) | 0.910 | 0.996 |  |  |
| Normal weight | Preterm birth | early pregnancy | Excessive | 1.00 (0.77, 1.29) | 0.998 | 0.998 |  |  |
| Normal weight | LBW | early pregnancy | Inadequate | 1.23 (0.91, 1.65) | 0.174 | 0.641 |  |  |
| Normal weight | LBW | early pregnancy | Excessive | 0.84 (0.65, 1.10) | 0.215 | 0.658 |  |  |
| Normal weight | Macrosomia | early pregnancy | Inadequate | 0.92 (0.48, 1.76) | 0.805 | 0.996 |  |  |
| Normal weight | Macrosomia | early pregnancy | Excessive | 1.55 (0.97, 2.48) | 0.066 | 0.373 |  |  |
| Normal weight | SGA | early pregnancy | Inadequate | 1.15 (0.94, 1.40) | 0.173 | 0.641 |  |  |
| Normal weight | SGA | early pregnancy | Excessive | 0.73 (0.61, 0.87) | **<0.001** | **0.011** | *** | † |
| Normal weight | LGA | early pregnancy | Inadequate | 0.89 (0.66, 1.20) | 0.437 | 0.809 |  |  |
| Normal weight | LGA | early pregnancy | Excessive | 1.27 (1.02, 1.59) | **0.034** | 0.236 | * |  |
| Normal weight | Placental abruption | early pregnancy | Inadequate | 0.85 (0.57, 1.27) | 0.426 | 0.809 |  |  |
| Normal weight | Placental abruption | early pregnancy | Excessive | 0.68 (0.48, 0.96) | **0.027** | 0.213 | * |  |
| Normal weight | Postpartum hemorrhage | early pregnancy | Inadequate | 0.87 (0.54, 1.42) | 0.589 | 0.883 |  |  |
| Normal weight | Postpartum hemorrhage | early pregnancy | Excessive | 1.08 (0.74, 1.57) | 0.687 | 0.931 |  |  |
| Normal weight | Preeclampsia | before OGTT | Inadequate | 0.91 (0.46, 1.80) | 0.785 | 0.996 |  |  |
| Normal weight | Preeclampsia | before OGTT | Excessive | 0.91 (0.63, 1.31) | 0.608 | 0.883 |  |  |
| Normal weight | Preterm birth | before OGTT | Inadequate | 1.54 (1.04, 2.28) | **0.032** | 0.233 | * |  |
| Normal weight | Preterm birth | before OGTT | Excessive | 1.16 (0.91, 1.48) | 0.241 | 0.694 |  |  |
| Normal weight | LBW | before OGTT | Inadequate | 1.71 (1.19, 2.45) | **0.004** | 0.061 | ** |  |
| Normal weight | LBW | before OGTT | Excessive | 0.72 (0.56, 0.92) | **0.009** | 0.100 | ** |  |
| Normal weight | Macrosomia | before OGTT | Inadequate | 0.37 (0.09, 1.58) | 0.180 | 0.641 |  |  |
| Normal weight | Macrosomia | before OGTT | Excessive | 2.06 (1.30, 3.27) | **0.002** | **0.039** | ** | † |
| Normal weight | SGA | before OGTT | Inadequate | 1.20 (0.91, 1.57) | 0.190 | 0.641 |  |  |
| Normal weight | SGA | before OGTT | Excessive | 0.67 (0.57, 0.79) | **<0.001** | **0.004** | *** | †† |
| Normal weight | LGA | before OGTT | Inadequate | 0.63 (0.38, 1.05) | 0.075 | 0.400 |  |  |
| Normal weight | LGA | before OGTT | Excessive | 1.71 (1.38, 2.13) | **<0.001** | **0.004** | *** | †† |
| Normal weight | Placental abruption | before OGTT | Inadequate | 1.05 (0.60, 1.86) | 0.856 | 0.996 |  |  |
| Normal weight | Placental abruption | before OGTT | Excessive | 0.97 (0.70, 1.33) | 0.835 | 0.996 |  |  |
| Normal weight | Postpartum hemorrhage | before OGTT | Inadequate | 0.55 (0.24, 1.29) | 0.169 | 0.641 |  |  |
| Normal weight | Postpartum hemorrhage | before OGTT | Excessive | 1.10 (0.78, 1.57) | 0.582 | 0.883 |  |  |
| Normal weight | Preeclampsia | after OGTT | Inadequate | 0.83 (0.50, 1.39) | 0.486 | 0.822 |  |  |
| Normal weight | Preeclampsia | after OGTT | Excessive | 4.06 (2.69, 6.12) | **<0.001** | **0.004** | *** | †† |
| Normal weight | Preterm birth | after OGTT | Inadequate | 1.37 (1.05, 1.79) | **0.020** | 0.171 | * |  |
| Normal weight | Preterm birth | after OGTT | Excessive | 1.79 (1.34, 2.40) | **<0.001** | **0.004** | *** | †† |
| Normal weight | LBW | after OGTT | Inadequate | 1.19 (0.91, 1.55) | 0.197 | 0.641 |  |  |
| Normal weight | LBW | after OGTT | Excessive | 1.35 (1.00, 1.82) | **0.050** | 0.309 | * |  |
| Normal weight | Macrosomia | after OGTT | Inadequate | 0.49 (0.29, 0.84) | **0.009** | 0.100 | ** |  |
| Normal weight | Macrosomia | after OGTT | Excessive | 1.05 (0.64, 1.74) | 0.838 | 0.996 |  |  |
| Normal weight | SGA | after OGTT | Inadequate | 1.04 (0.88, 1.24) | 0.629 | 0.890 |  |  |
| Normal weight | SGA | after OGTT | Excessive | 1.09 (0.89, 1.33) | 0.421 | 0.809 |  |  |
| Normal weight | LGA | after OGTT | Inadequate | 0.73 (0.58, 0.93) | **0.011** | 0.117 | * |  |
| Normal weight | LGA | after OGTT | Excessive | 1.20 (0.93, 1.55) | 0.153 | 0.641 |  |  |
| Normal weight | Placental abruption | after OGTT | Inadequate | 1.32 (0.94, 1.85) | 0.108 | 0.508 |  |  |
| Normal weight | Placental abruption | after OGTT | Excessive | 1.07 (0.70, 1.63) | 0.756 | 0.982 |  |  |
| Normal weight | Postpartum hemorrhage | after OGTT | Inadequate | 1.23 (0.84, 1.79) | 0.282 | 0.712 |  |  |
| Normal weight | Postpartum hemorrhage | after OGTT | Excessive | 1.04 (0.65, 1.66) | 0.865 | 0.996 |  |  |
| Overweight | Preeclampsia | early pregnancy | Inadequate | 1.06 (0.58, 1.91) | 0.852 | 0.996 |  |  |
| Overweight | Preeclampsia | early pregnancy | Excessive | 1.75 (1.09, 2.81) | **0.021** | 0.171 | * |  |
| Overweight | Preterm birth | early pregnancy | Inadequate | 1.28 (0.79, 2.09) | 0.321 | 0.742 |  |  |
| Overweight | Preterm birth | early pregnancy | Excessive | 1.25 (0.81, 1.92) | 0.322 | 0.742 |  |  |
| Overweight | LBW | early pregnancy | Inadequate | 1.14 (0.68, 1.90) | 0.625 | 0.890 |  |  |
| Overweight | LBW | early pregnancy | Excessive | 0.72 (0.44, 1.17) | 0.186 | 0.641 |  |  |
| Overweight | Macrosomia | early pregnancy | Inadequate | 0.93 (0.52, 1.67) | 0.815 | 0.996 |  |  |
| Overweight | Macrosomia | early pregnancy | Excessive | 1.21 (0.75, 1.97) | 0.433 | 0.809 |  |  |
| Overweight | SGA | early pregnancy | Inadequate | 1.26 (0.86, 1.84) | 0.238 | 0.694 |  |  |
| Overweight | SGA | early pregnancy | Excessive | 0.83 (0.58, 1.20) | 0.324 | 0.742 |  |  |
| Overweight | LGA | early pregnancy | Inadequate | 0.88 (0.62, 1.24) | 0.460 | 0.809 |  |  |
| Overweight | LGA | early pregnancy | Excessive | 1.21 (0.91, 1.61) | 0.193 | 0.641 |  |  |
| Overweight | Placental abruption | early pregnancy | Inadequate | 0.70 (0.33, 1.47) | 0.349 | 0.742 |  |  |
| Overweight | Placental abruption | early pregnancy | Excessive | 0.57 (0.29, 1.11) | 0.098 | 0.482 |  |  |
| Overweight | Postpartum hemorrhage | early pregnancy | Inadequate | 1.09 (0.49, 2.43) | 0.833 | 0.996 |  |  |
| Overweight | Postpartum hemorrhage | early pregnancy | Excessive | 1.55 (0.80, 2.99) | 0.197 | 0.641 |  |  |
| Overweight | Preeclampsia | before OGTT | Inadequate | 0.65 (0.32, 1.36) | 0.255 | 0.699 |  |  |
| Overweight | Preeclampsia | before OGTT | Excessive | 0.71 (0.46, 1.11) | 0.132 | 0.588 |  |  |
| Overweight | Preterm birth | before OGTT | Inadequate | 0.85 (0.42, 1.71) | 0.652 | 0.910 |  |  |
| Overweight | Preterm birth | before OGTT | Excessive | 1.23 (0.79, 1.92) | 0.354 | 0.742 |  |  |
| Overweight | LBW | before OGTT | Inadequate | 0.90 (0.45, 1.80) | 0.760 | 0.982 |  |  |
| Overweight | LBW | before OGTT | Excessive | 0.77 (0.48, 1.23) | 0.280 | 0.712 |  |  |
| Overweight | Macrosomia | before OGTT | Inadequate | 0.89 (0.36, 2.22) | 0.802 | 0.996 |  |  |
| Overweight | Macrosomia | before OGTT | Excessive | 1.96 (1.11, 3.46) | **0.020** | 0.171 | * |  |
| Overweight | SGA | before OGTT | Inadequate | 0.95 (0.58, 1.54) | 0.825 | 0.996 |  |  |
| Overweight | SGA | before OGTT | Excessive | 0.63 (0.45, 0.88) | **0.007** | 0.093 | ** |  |
| Overweight | LGA | before OGTT | Inadequate | 0.71 (0.42, 1.20) | 0.199 | 0.641 |  |  |
| Overweight | LGA | before OGTT | Excessive | 1.72 (1.26, 2.37) | **<0.001** | **0.017** | *** | † |
| Overweight | Placental abruption | before OGTT | Inadequate | 1.00 (0.47, 2.12) | 0.998 | 0.998 |  |  |
| Overweight | Placental abruption | before OGTT | Excessive | 0.28 (0.14, 0.55) | **<0.001** | **0.006** | *** | †† |
| Overweight | Postpartum hemorrhage | before OGTT | Inadequate | 2.01 (0.86, 4.73) | 0.109 | 0.508 |  |  |
| Overweight | Postpartum hemorrhage | before OGTT | Excessive | 1.07 (0.53, 2.16) | 0.858 | 0.996 |  |  |
| Overweight | Preeclampsia | after OGTT | Inadequate | 0.89 (0.45, 1.77) | 0.741 | 0.982 |  |  |
| Overweight | Preeclampsia | after OGTT | Excessive | 2.49 (1.46, 4.24) | **<0.001** | **0.017** | *** | † |
| Overweight | Preterm birth | after OGTT | Inadequate | 1.12 (0.68, 1.85) | 0.661 | 0.915 |  |  |
| Overweight | Preterm birth | after OGTT | Excessive | 1.24 (0.79, 1.96) | 0.347 | 0.742 |  |  |
| Overweight | LBW | after OGTT | Inadequate | 1.28 (0.67, 2.44) | 0.454 | 0.809 |  |  |
| Overweight | LBW | after OGTT | Excessive | 2.14 (1.22, 3.72) | **0.007** | 0.093 | ** |  |
| Overweight | Macrosomia | after OGTT | Inadequate | 0.79 (0.41, 1.49) | 0.463 | 0.809 |  |  |
| Overweight | Macrosomia | after OGTT | Excessive | 1.65 (0.98, 2.77) | 0.058 | 0.353 |  |  |
| Overweight | SGA | after OGTT | Inadequate | 1.27 (0.84, 1.93) | 0.258 | 0.699 |  |  |
| Overweight | SGA | after OGTT | Excessive | 1.26 (0.86, 1.84) | 0.240 | 0.694 |  |  |
| Overweight | LGA | after OGTT | Inadequate | 0.71 (0.49, 1.02) | 0.066 | 0.373 |  |  |
| Overweight | LGA | after OGTT | Excessive | 1.53 (1.13, 2.06) | **0.006** | 0.089 | ** |  |
| Overweight | Placental abruption | after OGTT | Inadequate | 1.12 (0.54, 2.29) | 0.766 | 0.982 |  |  |
| Overweight | Placental abruption | after OGTT | Excessive | 0.71 (0.34, 1.47) | 0.361 | 0.742 |  |  |
| Overweight | Postpartum hemorrhage | after OGTT | Inadequate | 1.35 (0.61, 2.97) | 0.458 | 0.809 |  |  |
| Overweight | Postpartum hemorrhage | after OGTT | Excessive | 1.50 (0.73, 3.08) | 0.272 | 0.706 |  |  |
| Obese | Preeclampsia | early pregnancy | Inadequate | 1.52 (0.81, 2.85) | 0.195 | 0.641 |  |  |
| Obese | Preeclampsia | early pregnancy | Excessive | 0.96 (0.51, 1.79) | 0.894 | 0.996 |  |  |
| Obese | Preterm birth | early pregnancy | Inadequate | 1.11 (0.55, 2.28) | 0.766 | 0.982 |  |  |
| Obese | Preterm birth | early pregnancy | Excessive | 1.02 (0.53, 1.97) | 0.959 | 0.996 |  |  |
| Obese | LBW | early pregnancy | Inadequate | 1.73 (0.75, 4.00) | 0.196 | 0.641 |  |  |
| Obese | LBW | early pregnancy | Excessive | 1.33 (0.59, 3.02) | 0.493 | 0.822 |  |  |
| Obese | Macrosomia | early pregnancy | Inadequate | 0.84 (0.35, 2.00) | 0.694 | 0.934 |  |  |
| Obese | Macrosomia | early pregnancy | Excessive | 1.08 (0.51, 2.28) | 0.842 | 0.996 |  |  |
| Obese | SGA | early pregnancy | Inadequate | 1.21 (0.59, 2.50) | 0.605 | 0.883 |  |  |
| Obese | SGA | early pregnancy | Excessive | 0.97 (0.48, 1.96) | 0.940 | 0.996 |  |  |
| Obese | LGA | early pregnancy | Inadequate | 0.86 (0.48, 1.57) | 0.631 | 0.890 |  |  |
| Obese | LGA | early pregnancy | Excessive | 1.56 (0.94, 2.59) | 0.085 | 0.441 |  |  |
| Obese | Placental abruption | early pregnancy | Inadequate | 2.01 (0.44, 9.30) | 0.371 | 0.753 |  |  |
| Obese | Placental abruption | early pregnancy | Excessive | 1.45 (0.31, 6.72) | 0.633 | 0.890 |  |  |
| Obese | Postpartum hemorrhage | early pregnancy | Inadequate | 1.07 (0.27, 4.16) | 0.927 | 0.996 |  |  |
| Obese | Postpartum hemorrhage | early pregnancy | Excessive | 1.15 (0.31, 4.31) | 0.835 | 0.996 |  |  |
| Obese | Preeclampsia | before OGTT | Inadequate | 0.67 (0.28, 1.57) | 0.355 | 0.742 |  |  |
| Obese | Preeclampsia | before OGTT | Excessive | 0.68 (0.38, 1.23) | 0.203 | 0.644 |  |  |
| Obese | Preterm birth | before OGTT | Inadequate | 1.49 (0.66, 3.38) | 0.337 | 0.742 |  |  |
| Obese | Preterm birth | before OGTT | Excessive | 0.66 (0.34, 1.29) | 0.228 | 0.687 |  |  |
| Obese | LBW | before OGTT | Inadequate | 1.21 (0.48, 3.10) | 0.684 | 0.931 |  |  |
| Obese | LBW | before OGTT | Excessive | 0.43 (0.20, 0.93) | **0.032** | 0.233 | * |  |
| Obese | Macrosomia | before OGTT | Inadequate | 1.25 (0.32, 4.83) | 0.748 | 0.982 |  |  |
| Obese | Macrosomia | before OGTT | Excessive | 2.43 (0.92, 6.44) | 0.073 | 0.400 |  |  |
| Obese | SGA | before OGTT | Inadequate | 1.24 (0.55, 2.81) | 0.604 | 0.883 |  |  |
| Obese | SGA | before OGTT | Excessive | 0.38 (0.19, 0.75) | **0.005** | 0.078 | ** |  |
| Obese | LGA | before OGTT | Inadequate | 0.99 (0.42, 2.35) | 0.988 | 0.998 |  |  |
| Obese | LGA | before OGTT | Excessive | 2.04 (1.12, 3.72) | **0.019** | 0.171 | * |  |
| Obese | Placental abruption | before OGTT | Inadequate | 0.87 (0.08, 9.99) | 0.913 | 0.996 |  |  |
| Obese | Placental abruption | before OGTT | Excessive | 1.52 (0.32, 7.31) | 0.598 | 0.883 |  |  |
| Obese | Postpartum hemorrhage | before OGTT | Inadequate | 3.51 (0.30, 41.21) | 0.318 | 0.742 |  |  |
| Obese | Postpartum hemorrhage | before OGTT | Excessive | 4.07 (0.51, 32.27) | 0.184 | 0.641 |  |  |
| Obese | Preeclampsia | after OGTT | Inadequate | 0.33 (0.11, 0.98) | **0.045** | 0.290 | * |  |
| Obese | Preeclampsia | after OGTT | Excessive | 1.23 (0.64, 2.39) | 0.535 | 0.856 |  |  |
| Obese | Preterm birth | after OGTT | Inadequate | 1.31 (0.47, 3.69) | 0.605 | 0.883 |  |  |
| Obese | Preterm birth | after OGTT | Excessive | 1.71 (0.74, 3.98) | 0.211 | 0.658 |  |  |
| Obese | LBW | after OGTT | Inadequate | 2.07 (0.59, 7.23) | 0.256 | 0.699 |  |  |
| Obese | LBW | after OGTT | Excessive | 1.77 (0.59, 5.26) | 0.307 | 0.742 |  |  |
| Obese | Macrosomia | after OGTT | Inadequate | 0.72 (0.25, 2.02) | 0.527 | 0.850 |  |  |
| Obese | Macrosomia | after OGTT | Excessive | 0.70 (0.31, 1.58) | 0.386 | 0.777 |  |  |
| Obese | SGA | after OGTT | Inadequate | 1.46 (0.52, 4.05) | 0.471 | 0.815 |  |  |
| Obese | SGA | after OGTT | Excessive | 1.37 (0.58, 3.23) | 0.475 | 0.815 |  |  |
| Obese | LGA | after OGTT | Inadequate | 1.01 (0.50, 2.04) | 0.977 | 0.998 |  |  |
| Obese | LGA | after OGTT | Excessive | 0.97 (0.55, 1.73) | 0.928 | 0.996 |  |  |
| Obese | Placental abruption | after OGTT | Inadequate | 1.08 (0.15, 7.85) | 0.942 | 0.996 |  |  |
| Obese | Placental abruption | after OGTT | Excessive | 1.05 (0.21, 5.23) | 0.950 | 0.996 |  |  |
| Obese | Postpartum hemorrhage | after OGTT | Inadequate | 3.02 (0.30, 30.84) | 0.350 | 0.742 |  |  |
| Obese | Postpartum hemorrhage | after OGTT | Excessive | 3.23 (0.40, 26.14) | 0.272 | 0.706 |  |  |

Multivariable logistic regression models were adjusted for maternal age, ethnicity, residence, gravidity, and parity. The Adequate GWG category served as the reference for each gestational stage.

Significance levels (unadjusted): * p < 0.05; ** p < 0.01; *** p < 0.001.

The Benjamini-Hochberg false discovery rate (FDR) correction was applied within each pre-pregnancy BMI stratum, encompassing six primary outcomes (preeclampsia, preterm birth, LBW, macrosomia, SGA, LGA) across three

gestational stages and two GWG categories (Inadequate and Excessive vs. Adequate reference).

Significance levels (FDR-adjusted): † q < 0.05; †† q < 0.01; ††† q < 0.001.

Cells reported as "—" indicate subgroups with zero events, where no logistic regression estimate could be reliably obtained.

##### Table S3. Statistical parameters of the Generalized Additive Models (GAM), including effective degrees of freedom (edf) and approximate p-values for the smooth terms of trimester-specific gestational weight gain.

| **BMI_group** | **Stage** | **Outcome** | **edf** | **p_value** |
| --- | --- | --- | --- | --- |
| Underweight | early pregnancy | Preeclampsia | 1.00 | 0.259 |
| Underweight | early pregnancy | Preterm birth | 1.00 | 0.684 |
| Underweight | early pregnancy | LBW | 1.00 | 0.677 |
| Underweight | early pregnancy | Macrosomia | 2.11 | 0.616 |
| Underweight | early pregnancy | SGA | 5.54 | 0.353 |
| Underweight | early pregnancy | LGA | 1.00 | 0.187 |
| Underweight | early pregnancy | Placental abruption | 2.83 | 0.299 |
| Underweight | early pregnancy | Postpartum hemorrhage | 1.00 | 0.160 |
| Underweight | before OGTT | Preeclampsia | 1.00 | 0.428 |
| Underweight | before OGTT | Preterm birth | 2.43 | **0.012** |
| Underweight | before OGTT | LBW | 2.15 | **0.041** |
| Underweight | before OGTT | Macrosomia | 2.20 | 0.676 |
| Underweight | before OGTT | SGA | 4.96 | **<0.001** |
| Underweight | before OGTT | LGA | 1.00 | 0.776 |
| Underweight | before OGTT | Placental abruption | 1.00 | 0.075 |
| Underweight | before OGTT | Postpartum hemorrhage | 1.00 | 0.445 |
| Underweight | after OGTT | Preeclampsia | 6.57 | 0.103 |
| Underweight | after OGTT | Preterm birth | 1.00 | 0.196 |
| Underweight | after OGTT | LBW | 1.23 | 0.603 |
| Underweight | after OGTT | Macrosomia | 1.00 | 0.542 |
| Underweight | after OGTT | SGA | 7.04 | 0.064 |
| Underweight | after OGTT | LGA | 1.00 | 0.125 |
| Underweight | after OGTT | Placental abruption | 1.00 | 0.686 |
| Underweight | after OGTT | Postpartum hemorrhage | 1.00 | 0.944 |
| Normal weight | early pregnancy | Preeclampsia | 2.79 | 0.520 |
| Normal weight | early pregnancy | Preterm birth | 1.00 | 0.731 |
| Normal weight | early pregnancy | LBW | 1.23 | **0.030** |
| Normal weight | early pregnancy | Macrosomia | 1.01 | **0.009** |
| Normal weight | early pregnancy | SGA | 1.00 | **<0.001** |
| Normal weight | early pregnancy | LGA | 5.77 | **<0.001** |
| Normal weight | early pregnancy | Placental abruption | 1.95 | 0.058 |
| Normal weight | early pregnancy | Postpartum hemorrhage | 1.00 | 0.458 |
| Normal weight | before OGTT | Preeclampsia | 1.00 | 0.410 |
| Normal weight | before OGTT | Preterm birth | 2.25 | 0.456 |
| Normal weight | before OGTT | LBW | 1.40 | **<0.001** |
| Normal weight | before OGTT | Macrosomia | 1.00 | **<0.001** |
| Normal weight | before OGTT | SGA | 1.01 | **<0.001** |
| Normal weight | before OGTT | LGA | 1.00 | **<0.001** |
| Normal weight | before OGTT | Placental abruption | 1.00 | 0.404 |
| Normal weight | before OGTT | Postpartum hemorrhage | 5.79 | 0.133 |
| Normal weight | after OGTT | Preeclampsia | 3.55 | **<0.001** |
| Normal weight | after OGTT | Preterm birth | 2.90 | **<0.001** |
| Normal weight | after OGTT | LBW | 3.13 | **<0.001** |
| Normal weight | after OGTT | Macrosomia | 3.72 | **0.009** |
| Normal weight | after OGTT | SGA | 1.77 | 0.309 |
| Normal weight | after OGTT | LGA | 1.01 | **<0.001** |
| Normal weight | after OGTT | Placental abruption | 3.38 | 0.066 |
| Normal weight | after OGTT | Postpartum hemorrhage | 1.01 | 0.160 |
| Overweight | early pregnancy | Preeclampsia | 1.00 | **0.035** |
| Overweight | early pregnancy | Preterm birth | 1.00 | 0.371 |
| Overweight | early pregnancy | LBW | 1.41 | 0.321 |
| Overweight | early pregnancy | Macrosomia | 1.01 | 0.196 |
| Overweight | early pregnancy | SGA | 1.00 | 0.061 |
| Overweight | early pregnancy | LGA | 1.86 | **0.017** |
| Overweight | early pregnancy | Placental abruption | 2.40 | 0.500 |
| Overweight | early pregnancy | Postpartum hemorrhage | 1.00 | **0.019** |
| Overweight | before OGTT | Preeclampsia | 2.77 | 0.215 |
| Overweight | before OGTT | Preterm birth | 1.00 | 0.395 |
| Overweight | before OGTT | LBW | 2.10 | 0.083 |
| Overweight | before OGTT | Macrosomia | 1.00 | **<0.001** |
| Overweight | before OGTT | SGA | 1.00 | **<0.001** |
| Overweight | before OGTT | LGA | 1.05 | **<0.001** |
| Overweight | before OGTT | Placental abruption | 3.43 | **0.003** |
| Overweight | before OGTT | Postpartum hemorrhage | 1.00 | 0.143 |
| Overweight | after OGTT | Preeclampsia | 1.83 | **<0.001** |
| Overweight | after OGTT | Preterm birth | 2.00 | 0.237 |
| Overweight | after OGTT | LBW | 6.81 | **<0.001** |
| Overweight | after OGTT | Macrosomia | 1.00 | **<0.001** |
| Overweight | after OGTT | SGA | 2.24 | 0.404 |
| Overweight | after OGTT | LGA | 1.32 | **<0.001** |
| Overweight | after OGTT | Placental abruption | 1.00 | 0.159 |
| Overweight | after OGTT | Postpartum hemorrhage | 1.00 | 0.667 |
| Obese | early pregnancy | Preeclampsia | 1.94 | 0.149 |
| Obese | early pregnancy | Preterm birth | 1.00 | 0.351 |
| Obese | early pregnancy | LBW | 5.95 | 0.239 |
| Obese | early pregnancy | Macrosomia | 1.00 | 0.197 |
| Obese | early pregnancy | SGA | 1.00 | 0.366 |
| Obese | early pregnancy | LGA | 1.26 | **0.011** |
| Obese | early pregnancy | Placental abruption | 1.53 | 0.597 |
| Obese | early pregnancy | Postpartum hemorrhage | 1.00 | 0.896 |
| Obese | before OGTT | Preeclampsia | 1.00 | 0.987 |
| Obese | before OGTT | Preterm birth | 1.94 | 0.119 |
| Obese | before OGTT | LBW | 5.03 | 0.078 |
| Obese | before OGTT | Macrosomia | 1.63 | 0.241 |
| Obese | before OGTT | SGA | 1.76 | **0.005** |
| Obese | before OGTT | LGA | 1.00 | **0.001** |
| Obese | before OGTT | Placental abruption | 1.00 | 0.061 |
| Obese | before OGTT | Postpartum hemorrhage | 1.00 | 0.275 |
| Obese | after OGTT | Preeclampsia | 1.26 | **<0.001** |
| Obese | after OGTT | Preterm birth | 2.30 | **0.011** |
| Obese | after OGTT | LBW | 1.89 | 0.320 |
| Obese | after OGTT | Macrosomia | 2.38 | 0.206 |
| Obese | after OGTT | SGA | 1.40 | 0.666 |
| Obese | after OGTT | LGA | 1.72 | 0.192 |
| Obese | after OGTT | Placental abruption | 1.00 | 0.665 |
| Obese | after OGTT | Postpartum hemorrhage | 1.00 | 0.354 |
